# Assessment of Angiogenesis and Cell Survivability of an Inkjet Bioprinted Biological Implant in an Animal Model

**DOI:** 10.3390/ma15134468

**Published:** 2022-06-24

**Authors:** Beu P. Oropeza, Carlos Serna, Michael E. Furth, Luis H. Solis, Cesar E. Gonzalez, Valeria Altamirano, Daisy C. Alvarado, Jesus A. Castor, Jesus A. Cedeno, Dante Chaparro Vega, Octavio Cordova, Isaac G. Deaguero, Erwin I. Delgado, Mario F. Garcia Duarte, Mirsa Gonzalez Favela, Alba J. Leyva Marquez, Emilio S. Loera, Gisela Lopez, Fernanda Lugo, Tania G. Miramontes, Erik Munoz, Paola A. Rodriguez, Leila M. Subia, Arahim A. Zuniga Herrera, Thomas Boland

**Affiliations:** 1 Department of Metallurgy, Materials, and Biomedical Engineering, The University of Texas at El Paso, El Paso, TX 79968, USA; boropeza@utep.edu (B.P.O.); carlos.iii@northwestern.edu (C.S.III); mefurth@miners.utep.edu (M.E.F.); lhsolis@miners.utep.edu (L.H.S.); valtamirano@utep.edu (V.A.); dcalvarado2@miners.utep.edu (D.C.A.); jacastor@miners.utep.edu (J.A.C.); jacedeno2@miners.utep.edu (J.A.C.); dchaparro2@miners.utep.edu (D.C.V.); ocordovajr@miners.utep.edu (O.C.); isaac.deaguero@va.gov (I.G.D.); eidelgado2@miners.utep.edu (E.I.D.); mfgarcia5@miners.utep.edu (M.F.G.D.); mgonzalezfavela@miners.utep.edu (M.G.F.); ajleyva@miners.utep.edu (A.J.L.M.); esloera@miners.utep.edu (E.S.L.); glopez40@miners.utep.edu (G.L.); flugo@miners.utep.edu (F.L.); tgmiramontes@miners.utep.edu (T.G.M.); emunoz17@miners.utep.edu (E.M.); parodriguez11@miners.utep.edu (P.A.R.); lmsubia@miners.utep.edu (L.M.S.); azunigaherrera@miners.utep.edu (A.A.Z.H.); 2 Department of Chemistry and Biochemistry, The University of Texas at El Paso, El Paso, TX 79968, USA; cegonzalez14@miners.utep.edu

**Keywords:** tissue engineering, bio-printing, microvasculature, angiogenesis, inkjet printing

## Abstract

The rapidly growing field of tissue engineering hopes to soon address the shortage of transplantable tissues, allowing for precise control and fabrication that could be made for each specific patient. The protocols currently in place to print large-scale tissues have yet to address the main challenge of nutritional deficiencies in the central areas of the engineered tissue, causing necrosis deep within and rendering it ineffective. Bioprinted microvasculature has been proposed to encourage angiogenesis and facilitate the mobility of oxygen and nutrients throughout the engineered tissue. An implant made via an inkjet printing process containing human microvascular endothelial cells was placed in both B17-SCID and NSG-SGM3 animal models to determine the rate of angiogenesis and degree of cell survival. The implantable tissues were made using a combination of alginate and gelatin type B; all implants were printed via previously published procedures using a modified HP inkjet printer. Histopathological results show a dramatic increase in the average microvasculature formation for mice that received the printed constructs within the implant area when compared to the manual and control implants, indicating inkjet bioprinting technology can be effectively used for vascularization of engineered tissues.

## 1. Introduction

Over the last two decades, new methods for processing biodegradable polymeric materials with customizable physico-chemical structural features have driven the field of biomedical engineering [1]. In particular, the evolution of two-dimensional (2D) printing into ‘layer upon layer’ additive manufacturing (AM), or three-dimensional (3D) printing, has had considerable implications for tissue engineering (TE) [1,2]. In its nascence, the concept of culturing organ tissues for transplantation was realized using biodegradable or bioerodable scaffolds to support individual cell seeding [3]. In 3D bioprinting, a ‘bioink’ is dispensed onto or into a processible scaffold to form mechanically supportive structures containing bioactive or cellular components [4,5]. To avoid the drying of cells, these scaffolds are typically composed of water-retaining soft materials such as hydrogels. The biocompatibility of alginate and its ability to crosslink in the presence of calcium ions makes it an ideal material for the composition of these temporary templates [6]. Additionally, these hydrogel constructs provide cells with an initial reservoir of nutrients and an environment conducive to promotive diffusion [7]. However, there remains a challenge in providing bioengineered constructs with an adequate supply of nutrients and oxygen post-implantation [8].

As the thickness of engineered tissues exceeds 150–200 µm, the oxygen diffusion threshold of the host vasculature is surpassed, and provisions become limited [9]. To resolve this issue, tissue engineers must incorporate sufficient vasculature within the engineered tissue to maintain cell viability and function [10]. Thermal inkjet-based bioprinting (TIB) is one promising approach to developing vasculature or microvasculature systems for complex multicellular constructs. Thermal inkjet printing was the first technology repurposed for bioprinting applications and remains one of today’s primary approaches [11]. DNA chips and protein arrays created using modified commercial inkjet printers were among the first products of biomolecular printing [12,13,14]. The following success in seeding viable bacteria and mammalian cells demonstrated the potential of TIB for TE applications [14,15].

In TIB, digital data from a computer is translated into intermittent bursts of ink onto a ‘biopaper’. The reproduction of digital patterns using computer software is advantageous in providing control over ejection points. In addition, using inkjet printing minimizes the risk of contamination due to the separation, or non-contact, between the cartridge nozzles and printing substrate [16]. Moreover, because TIB uses modified commercial printers and cartridges, it is low in cost, has high throughput, and is capable of operating at high frequency [9,16]. Once a bioink is loaded into a cartridge, droplets are ejected using pressure pulses from collapsing air bubbles; in this way, drops are generated ‘on-demand’ [17]. Despite somewhat unknown conditions inside the printhead assembly, ejected cells have been shown to survive with an average viability of 90% [18]. 

Endothelial cells printed using TIB have been observed to proliferate into confluent lining, precursors to microvasculature formation [6,19]. Furthermore, it has been proposed that the TIB of endothelial cells induces angiogenic pathways such as the NF-κB to produce VEGF, as we have shown elevated levels of cytokines that induce this pathway. [20]. TIB can be used with great precision, with studies showing that droplets can eject as little as one cell at a time [21,22]. This precision is prime for microvascular engineering, where a small number of cells should be deposited at a time to form the small vessels. Other advantages of TIB that have previously been found are high cell viability, high printing speed, low manufacturing cost, and high reproducibility of the print [16,23]. Using an animal model, this study further examines the impact of vascular and elongation factors released during TIB. We hypothesize that using TIB to seed endothelial cells onto an alginate/gelatin scaffold will improve microvasculature formation compared to manual seeding in vivo, leading to the ability to engineer thick tissues for medical implantations.

## 2. Materials and Methods

### 2.1. Animal Experiments

This protocol was approved by the Institutional Animal Care and Use Committee (IACUC) at The University of Texas at El Paso with protocol number A-201701-4-1009030-1; date of approval: 12/4/2019 (renewal date). A total of forty-eight female B17-SCID and NSG-SGM3 mice were purchased from Jackson Laboratory and were kept under barrier conditions at the animal housing facility in The Border Biomedical Research center at The University of Texas at El Paso.

### 2.2. Cell Culture

Human Microvascular Endothelial Cells (HMVECs) from a commercial source (Lonza Bioscience, Morristown, NJ, USA CC 2505) were grown in EGM-2 plus bullet kit (CC-3162, Lonza Bioscience) under humidified culture conditions at 37 °C with 5% CO_2_ in a fully aseptic environment.

### 2.3. Hydrogel Preparation

In this study, a composite hydrogel was prepared with 2% (*w*/*v*) alginic acid (Thermo Fisher Scientific, Waltham, MA, USA) and 5% gelatin type B (Thermo Fisher Scientific) by dissolving the appropriate amounts in PBS and autoclaving the solution. Crosslinking was achieved during the inkjet printing process with 0.15M of CaCl_2_ (Thermo Fisher Scientific), as described elsewhere [19].

### 2.4. Bioprinting

Vascular and mock implant printing was carried out using a modified HP inkjet printer [21]. Mock implants contained no cells but were otherwise identical in size and composition. TIB implants were made using trypsinized, detached, and neutralized HMVECs resuspended in a 0.15M CaCl_2_ solution for a final cell concentration of 4 × 10^6^ cells/mL to form a low viscosity bioink. This bioink was then printed atop a mixture of 2% alginic acid and 5% gelatin. As bioprinting of the bioink occurs, crosslinking of the alginic acid is seen, and the resulting hydrogel is further stabilized by immersing the entire gel in 0.15M CaCl_2_ solution for 15 min. Manual implants were implants containing cells from the same batch of cells as the TIB implants. These cells were suspended in the same bioink as the cells for the TIB implants but manually seeded onto the scaffolds. These scaffolds were also strengthened by immersing them in 0.15M CaCl_2_ solution for 15 min. All samples were stored in the endothelial growth medium in the incubator for approximately 12–15 h prior to implantation.

### 2.5. Surgical Procedure

**Figure 1:**
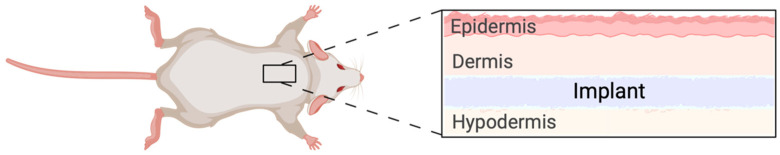
Schematic representation of implant placement in the in vivo model. The implant was inserted in a subcutaneous pocket in the dorsal thoracic area. The placement ensured the animal was not able to easily access the wound and cause harm to the area that was being monitored.

For surgical procedures the surgeon wore standard PPE to include sterile surgical gloves. Surgery was performed in a freshly decontaminated biosafety cabinet. Surgical instruments were autoclaved prior to use, and the surgeon will adhered to IACUC Policy 004 for all survival surgeries. Animals were continuously monitored with thermal support under half the recovery cage until return of the righting reflex (within 15 min); they were then placed in a sterilized cage with sterile, surgical bedding (diamond chip—Shepherd Specialty Paper Company). For pain management, mice received a SC injection of buprenorphine (0.05–0.1 mg/kg) prior to induction via vaporized isoflurane 4–5% in an induction chamber to achieve a surgical plane of anesthesia. Post-operatively the animals received buprenorphine injections if there were signs of pain as evidenced by posture, grimace or other indicators. Monitoring continued on an at least a once daily basis until experimental endpoints were reached. A postoperative monitoring sheet was completed at each check for every animal. A 1 × 1 cm dorsal thoracic subcutaneous pocket was made, the implant was placed in the prepared site (Figure 1), and the incision was closed using a non-degradable suture. Animals were continuously monitored with thermal support under half the recovery cage until the righting reflex was returned; subjects were then placed in a sterilized cage with sterile surgical bedding. Animals were placed on antibiotic feed for three days post-operatively; the sutures were removed five days post-operatively. Animals were euthanized for tissue collection via vaporized 5% isoflurane in an induction chamber six weeks post-operatively. At that point, a 2 cm × 2 cm × 0.5 cm area was excised, which included the original implant site as well as the surrounding tissues. All samples were fixed using 4% paraformaldehyde (Thermo Fisher Scientific) for 24 h before being processed.

### 2.6. Histology

The tissue samples were processed using an automated tissue-processing machine (Thermo Scientific Spin Tissue Processor Microtome STP-120). Tissue dehydration was achieved by immersing tissue samples in different concentrations of ethanol (starting with 70%, 95%, 100%) (Decon Laboratories Inc., King of Prussia, PA, USA), followed by immersion in xylene (Fisher Chemical) two times and paraffin infiltration. Paraffin-embedded tissues were sectioned to 4–6 μm Shandon Finesse E/ME microtome. All samples were deparaffinized in three xylene washes and rehydrated in decreasing ethanol concentrations (100%, 95%, 70%, 50%), with each wash lasting 3 min. Hematoxylin and eosin (H&E) (BBC Biochemical) staining was carried out post-deparaffinization. Following rehydration, the samples were exposed to hematoxylin solution for 2 min, then rinsed with running water for 1 min, distilled water for 30 s, and 95% alcohol for 30 s. Immediately after, the samples were exposed to eosin solution for 1 min, then washed with increasing amounts of alcohol (95% ×2, 100% ×2) for 2 min each, then two xylene washes each for 2 min. Trichrome staining was carried out using Masson’s Trichrome Staining Kit; all kit procedures were precisely followed. All samples were mounted using Cytoseal 60 (Thermo Scientific REF#8310-4). All procedures were taken from previously published protocols [24].

### 2.7. Immunofluorescence

Sections for immunofluorescence were deparaffinized and rehydrated using the protocol described above. Antigen unmasking was performed by covering samples in 1 mM EDTA and heating until the buffer boiled, followed by a cooling period of 20 min; sections were then incubated in two washes of 1× PBS each for 5 min, and one wash of 1× PBS supplemented with freshly made sodium borohydride (10 mg/mL, Sigma-Aldrich) for 40 min followed by four washes of 1× PBS each for 5 min. The sections were then permeabilized by incubating the sections in one was of 1× PBS supplemented with 0.2% Triton X-100 (Sigma-Aldrich) for 45 min, followed by three washes in 1× PBS for 5 min each. Staining of the sections was completed by incubating sections in 1× PBS supplemented with 1% blocking buffer (Invitrogen) for 1 h, followed by overnight incubation of 1× PBS with 1% blocking buffer and primary antibodies (CD31, Abcam) at 4 °C. The sections were washed three times with 1× PBS for 5 min each, then incubated in 1× PBS supplemented with the secondary antibody (Mouse IgG (Anti-mouse) Goat Alexa Flour 568 (2 µg/mL, Abcam)) for 1 h at room temperature followed by three additional washes in 1× PBS for 5 min, incubation of 10 min in 1× PBS supplemented with DAPI (0.5 μg/mL, Abcam), and washing twice with 1× PBS for 5 min each. All samples were mounted using Cytoseal 60 (Thermo Scientific REF#8310-4) and were stored at 4 °C in the dark. Image J was used to compare the amount of CD31 in the control samples versus what was seen in the printed and manual samples.

### 2.8. Statistical Analysis

Sections for immunofluorescence were scored blindly by three researchers. The significance of the difference in independent variables was determined by *p*-values of <0.05. A comparison of the two variables was made using a two-tailed sample *t*-test.

## 3. Results

### 3.1. Histopathological Analysis

**Figure 2:**
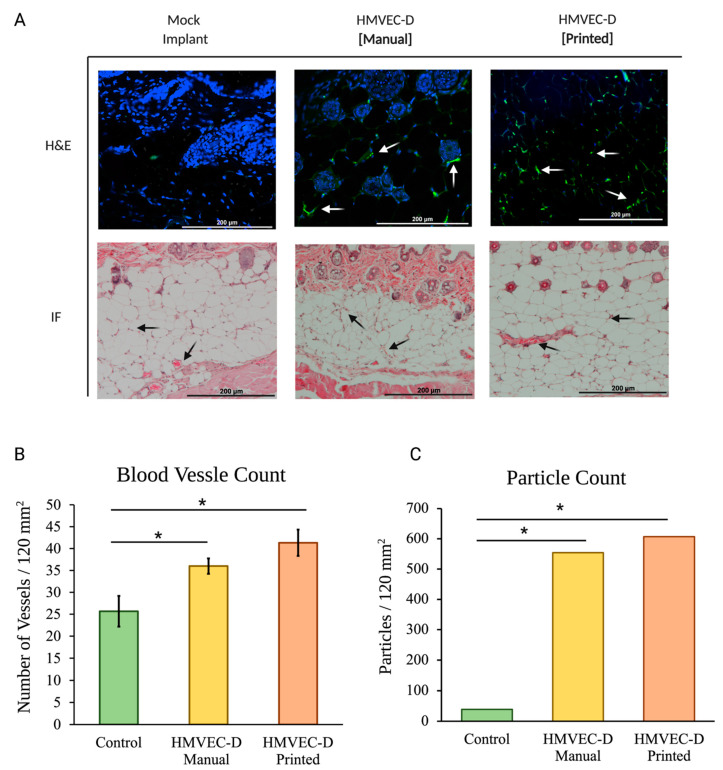
(**A**) In vivo tissue sections of the B17-SCID mice. Hematoxylin, eosin, and immunohistochemistry staining (blue-DAPI, green-CD31) visualize engineered vessels. Arrows indicate microvascular formations. (**B**) Blood vessel quantification of TIB implants show a 1.4× increase in the number of blood vessels per area compared to the manually made mock implants. The inkjet-printed implants presented 1.77× more vessels than printed mock implants. (**C**) Particle analysis of CD31-stained tissue samples show a 14× and 15× increase for the manually seeded and TIB samples, respectively. * denotes statistical significance *p* < 0.05.

Figure 2A shows the H&E and immunofluorescence of sections from the mock, the manually seeded, and the TIB scaffolds implanted in the B17-SCID mouse.

TIB implants show a 1.4× increase in the number of blood vessels per area compared to the manually made mock implants. The inkjet printed implants presented 1.77× more vessels than printed mock implants, as seen in Figure 2B.

**Figure 3:**
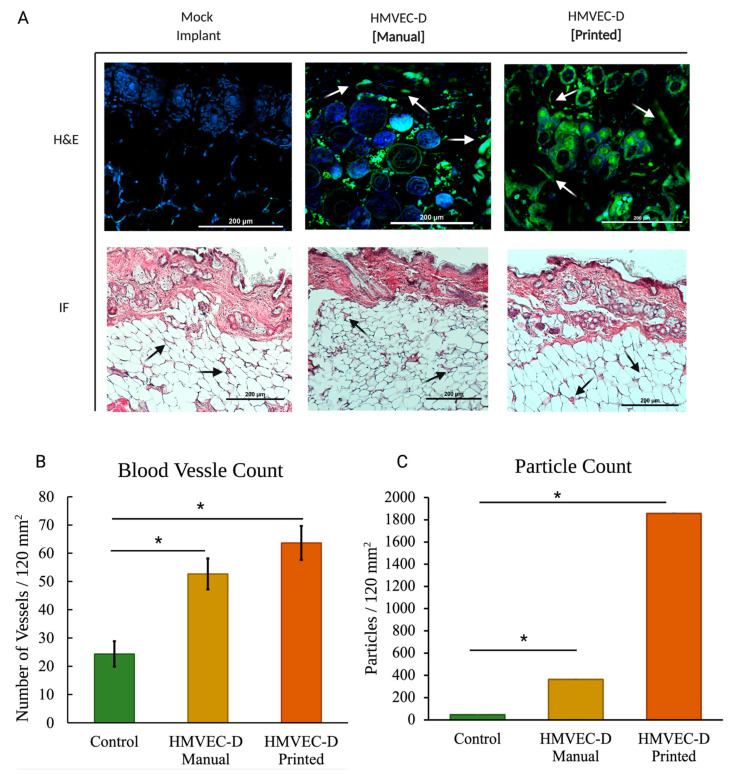
(**A**) In vivo vascular formation for the NSG-SMG3 mice. Hematoxylin, eosin, and immunohistochemistry-stained (blue-DAPI, green-CD31). Arrows indicate microvascular formation. (**B**) For blood vessel quantification of NSG-SGM3 mice, we observed more than twice the amount of vessels when comparing the inkjet-printed implants to those from the mock control group; specifically, a 2.16 increase in vessels per area was counted. Similarly, 2.89× of vessels were seen for TIB implants vs. manually seeded implants in the NSG-SGM3. (**C**) Particle analyses of the CD31-stained NSG-SMG3 tissue sections display a 7.8× increase for the manually seeded cells when compared to the control group and a 40× increase when compared to the TIB group. * denotes statistical significance *p* < 0.05.

**Figure 4:**
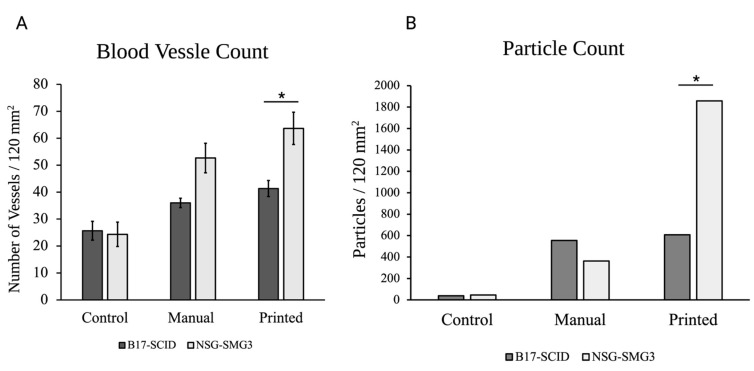
A comparison of the average number of vasculatures (**A**) and particle count for the CD31 stained sections (**B**) seen throughout the printed and manually seeded tissue sections for both types of mice (* *p* < 0.05, *n* = 3). All numbers were observed in 120 mm^2^ tissue sections.

Figure 3 shows the H&E and immunofluorescence of sections from the mock, the manually seeded, and the TIB scaffolds implanted in the NSG-SGM3 mouse. For NSG-SGM3 mice, we observed more than twice the amount of vessels when comparing the inkjet-printed implants to those from the mock control group; specifically, a 2.16 increase in vessels per area was counted. Similarly, 2.89× of vessels were seen for TIB implants vs. manually seeded implants in the NSG-SGM3 (see Figure 3). Additionally, for printed implants in the NSG-SMG3 model, a 1.5 increase in the vessel amount was observed compared to the same implant in a B17-SCID model (Figure 4A). All of the above comparisons were significant with a *p* ≤ 0.05.

### 3.2. Immunohistochemical Analysis

The CD31 stain displays the presence of the microvascular endothelial cells within the tissue samples. Cell-laden samples display greater interactions of the engineered blood vessels, particularly those in the inkjet-printed B17-SCID model (Figure 2), indicating the occurrence of angiogenesis throughout the tissue sample. Particle analysis comparison between the samples shows a 14× increase between the manually seeded and control groups, with the numbers enlarging to 15× for the TIB and the control group (Figure 2C).

NSG-SGM3 tissue samples showed a greater prevalence of the HMVECs throughout the sample (Figure 3). Additionally, the number of visible vessels increases when the implant was made through inkjet printing methods for both models. Figure 4 shows a comparative quantitation of the results within a 120 mm^2^ area of each tissue section. The particle analysis between the NSG_SMG3 samples and control groups showed a great difference, with the manually seeded implants showing a 7.8× increase and 40× increase for the TIB samples.

## 4. Discussion

**Figure 5:**
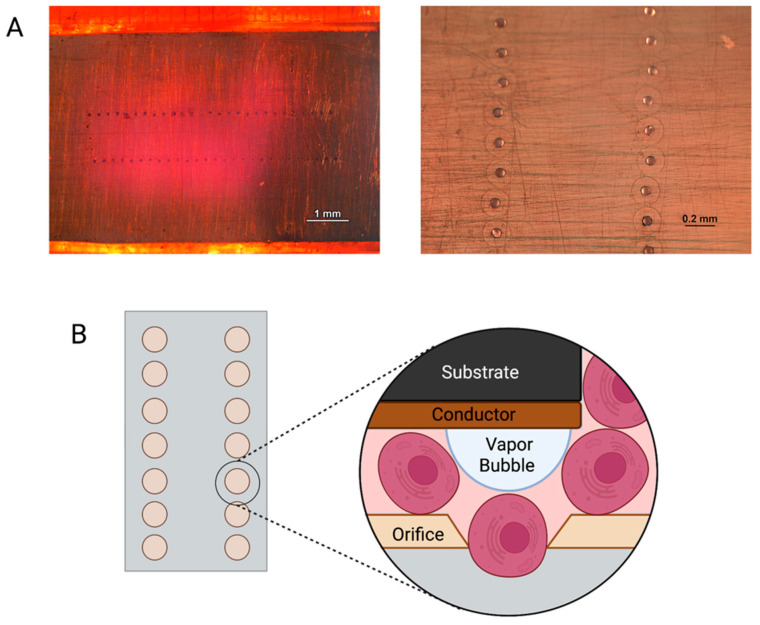
(**A**) Images of inkjet printer cartridge nozzles under a light microscope. (**B**) Schematic representation of cells being printed through an inkjet nozzle.

**Figure 6:**
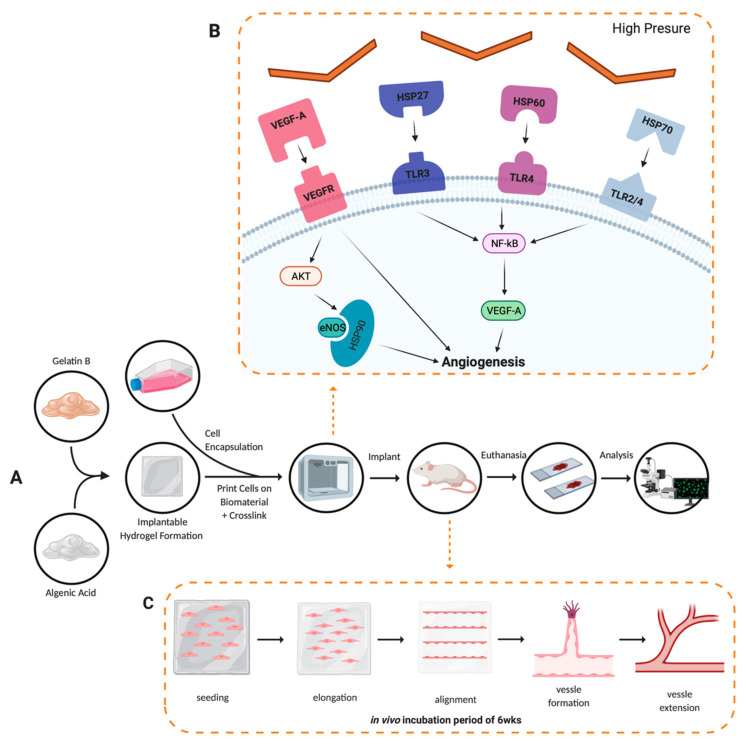
Angiogenesis development conceptual model. (**A**) Experimental procedure followed throughout the project, solid black arrows represent the observable progression of the project; dashed orange arrows denote a theoretical representation of angiogenic development. (**B**) Cellular pathway activation by thermal inkjet printing technologies, droplets of bioink are heated, binding VEGF-A to VEGFR, HSP27 to TLR3, HSP60 to TLR4, and HSP70 to TLR2/4, all leading to the angiogenesis seen in the analysis of tissues procured [20]. (**C**) Alginate/gelatin hydrogel degradation and vessel formation of the human microvascular endothelial cells via the inkjet-printing process.

Thermal inkjet printing inherently causes harsh conditions via temperature and pressure spikes to be enacted upon the cells throughout the bioprinting process [18]. The cells are forced out of a nozzle with an orifice approximately 70 μm in diameter, see Figure 5. That is larger than the average diameter of a cell strainer used to separate individual cells from a suspended endothelial cell solution, which is 40 μm in diameter. Nevertheless, we have previously seen membrane effects of TIB cells [18]. Previous in vitro work by Solis et al. found significant overexpression of many cytokines promoting endothelial cell growth. IL-8, IL-1α, HSP70, and VEGF-A were significantly overexpressed in TIB cells. The overexpression of HSP70 confirmed the elicitation of heat shock response. IL-1α increased over three times compared to media alone, confirming the cell-based trauma response [25]. IL-1α is known to stimulate the secretion of HSP70 family proteins, further amplifying the effect [26]. The expression of VEGF-A was more than doubled among TIB cells compared to media alone, which contains large amounts of this cytokine. We attributed this to the effects that HSP70 expression have on VEGF production [27]. IL-8, which is a pro-angiogenic cytokine [28], increasing proliferation, migration, and angiogenesis [29,30] by inducing VEGF secretion, was almost 700 times more expressed for TIB cells than in media alone. These in vitro findings suggest that, amongst others, the strongest pro-angiogenic cytokine, VEGF-A, will also be released into the scaffolds before and after implantation into the animals, causing cell elongation and vessel formation [31,32]. The elongation caused by TIP and stressors acting on the cells’ membranes throughout the printing processes appears to trigger the angiogenic cascade. This process appears to continue in the animals. This upregulation of VEGF-A and the overexpression of related pro-angiogenic cytokines seen in the in vitro studies appear to cement the significant increase in the number of vessels seen in our in vivo models. A schematic of this process is shown in Figure 6.

Initially, the engineered implant was placed in a B17-SCID animal model in order to reduce the possibilities of rejection, given that primary human endothelial cells were used. The significant increase of vessels observed throughout the region, coupled with the lack of structural changes seen in the surrounding tissues, led to the determination of successfully engineering microvasculature. In order to mimic the fate of an allograft in humans, we also observed the engineered implants within the NSG-SGM3 humanized mice model. Previous studies with this model have shown graft rejection which included near-complete loss of the vasculature and destruction of the dermal and epidermal layers [33] when HLA mismatched cells were used in the grafts. Additionally, the ability to achieve high engraftment of human cells within the model and the number of studies that have found rejection of human skin allografts when using NSG mice indicate the model’s applicability to determine implant rejection [34,35].

Our experiments revealed an even greater increase in vessel numbers for those with an engineered implant when compared to the mock implant in the same model and a significant increase (1.5×) compared to the printed implant placed in the B17-SCID model. While we did not observe structural changes or necrosis at the implant site or surrounding tissue, we assume that the leucocytes of the humanized mice must have killed the endothelial cells within the scaffolds. The increased amount of blood vessels seen in the model is most likely due to mouse endothelial cells being activated by the cytokines released from the scaffold, which may have been amplified by the lysing of the human cells in the implant. If this hypothesis is correct, one could envision the translation of this technique to humans in cases where increased vascularization is warranted.

Additionally, the observed microvasculature was determined based on the appearance of red blood cells. This indicates that the increased vessels were functional, and anastomosis occurred between the original native tissue. Given the oxygen diffusion problems that the tissue engineering community has long faced, the ability to trigger angiogenesis via the thermal inkjet-printing process would help build thicker tissues, which could lead to addressing the shortage of biological tissues used for surgical interventions.

## 5. Conclusions

These studies show that native vasculature can be augmented by implanting a TIB engineered scaffold. The inkjet-printing approach causes many pro-angiogenic factors to be released by the printed endothelial cells. In turn, this causes the new vessels to develop near the implant site, which join with previously existing ones to allow for blood circulation. The activation of angiogenic pathways by TIB appears to initiate post-implantation effects, which include a significantly increased microvasculature in the host. The TIB method could be a functional alternative to current methods of creating vascular networks within in vivo engineered tissues.

## Data Availability

Data are available upon request to the corresponding author or at the following link: https://minersutep-my.sharepoint.com/:f:/g/personal/tboland_utep_edu/EtFZlq_pcP9Kn1aNkeVcwO4Bu1KUMbBYKgSR-_8u53dkJw?e=vRmOtn.
